# Dual usage of a stage-specific fluorescent reporter system based on a helper-dependent adenoviral vector to visualize osteogenic differentiation

**DOI:** 10.1038/s41598-019-46105-y

**Published:** 2019-07-04

**Authors:** Takefumi Sone, Masashi Shin, Takehito Ouchi, Hiroki Sasanuma, Arei Miyamoto, Satoshi Ohte, Sho Tsukamoto, Mahito Nakanishi, Hideyuki Okano, Takenobu Katagiri, Kohnosuke Mitani

**Affiliations:** 1 Division of Gene Therapy and Genome Editing, Research Center for Genomic Medicine, Saitama Medical University, Hidaka, Saitama 350-1241 Japan; 2 Division of Pathophysiology, Research Center for Genomic Medicine, Saitama Medical University, Hidaka, Saitama 350-1241 Japan; 3 Department of Dentistry and Oral Surgery, Keio University School of Medicine, 35 Shinanomachi, Shinjuku-ku, Tokyo, 160-8582 Japan; 4 Biotechnology Research Institute for Drug Discovery, National Institute of Advanced Industrial Science and Technology (AIST), Central 5, 1-1-1 Higashi, Tsukuba, Ibaraki 305-8565 Japan; 5 Department of Physiology, Keio University School of Medicine, 35 Shinanomachi, Shinjuku-ku, Tokyo 160-8582 Japan; 6 Present Address: Takara Bio Inc., 7-4-38 Nojihigashi, Kusatsu, Shiga 525-0058 Japan; 7 Present Address: Department of Physiological Science and Molecular Biology, Division of Biomedical Sciences, Fukuoka Dental College, 2-15-1 Tamura, Sawara-ku, Fukuoka 814-0193 Japan; 8 Present Address: Laboratory Animal Research Center, The Institute of Medical Science, The University of Tokyo, 4-6-1 Shirokanedai, Minato-ku, Tokyo 108-8639 Japan; 9 Present Address: Department of Oral Science, Graduate School of Dentistry, Kanagawa Dental University, 82 Inaoka-cho, Yokosuka, Kanagawa 238-8580 Japan; 10 Present Address: Department of Microbial Chemistry, Graduate School of Pharmaceutical Sciences, Kitasato University, 5-9-1 Shirokane, Minato-ku, Tokyo 108-8641 Japan

**Keywords:** Fluorescence imaging, Bone development

## Abstract

We developed a reporter system that can be used in a dual manner in visualizing mature osteoblast formation. The system is based on a helper-dependent adenoviral vector (HDAdV), in which a fluorescent protein, Venus, is expressed under the control of the 19-kb human *osteocalcin* (*OC*) genomic locus. By infecting human and murine primary osteoblast (POB) cultures with this reporter vector, the cells forming bone-like nodules were specifically visualized by the reporter. In addition, the same vector was utilized to efficiently knock-in the reporter into the endogenous *OC* gene of human induced pluripotent stem cells (iPSCs), by homologous recombination. Neural crest-like cells (NCLCs) derived from the knock-in reporter iPSCs were differentiated into osteoblasts forming bone-like nodules and could be visualized by the expression of the fluorescent reporter. Living mature osteoblasts were then isolated from the murine mixed POB culture by fluorescence-activated cell sorting (FACS), and their mRNA expression profile was analyzed. Our study presents unique utility of reporter HDAdVs in stem cell biology and related applications.

## Introduction

Osteoblasts are specialized cells for bone formation and are derived from undifferentiated mesenchymal progenitor cells. Osteoblasts secrete organic extracellular matrices, such as type I collagen (COLI), osteopontin (SPP1), osteonectin (SPARC), bone sialoprotein (BSP) and osteocalcin (OC), to form the unmineralized bone matrix known as osteoid^1^. OC is one of the most abundant non-collagenous proteins in the bone matrix and has a high affinity for hydroxyapatite through gamma-carboxylated glutamic acids^2^. *OC* is a unique gene specifically expressed in bone-forming osteoblasts in the bone marrow cavity. In primary osteoblasts (POBs) prepared from bone tissues, the expression levels of *OC* mRNA gradually increase, in parallel with an increase of bone-like nodule formation^1,2^. Bone-specific transcription factor Runx2 interacts with the calcitropic hormone, 1α,25(OH)_2_D_3_ receptor, to up-regulate the rat *OC* gene expression in osteoblastic cells through the vitamin D receptor regulatory elements (VDREs) found in the 5′ upstream elements of the *OC* gene^3^. Today, the expression of OC is widely used as a specific marker of mature osteoblasts. Previously, a GFP transgenic mouse line driven by a 3.8-kb fragment of human *OC* promoter was used to show the mature osteoblast-specific expression of GFP^4^. A similar 3.8-kb *OC* promoter-GFP construct was transiently introduced into human bone marrow-derived cells by nucleofection to visualize osteogenic differentiation^5,6^. A luciferase reporter driven by the 10-kb human *OC* enhancer/promoter was used to visualize the bone formation of transgenic mice^7^. The same construct was inserted into a mammalian artificial chromosome (MAC) and transferred into human mesenchymal stem cells via chromosome transfer to monitor osteogenic differentiation^8^.

In the present study, we developed a novel system using a high-capacity helper-dependent adenoviral vector (HDAdV), in which Venus, an enhanced yellow fluorescent protein^9^, is expressed under control of the 19-kb human *OC* locus (HDAd-hOC-Venus). The HDAdV-based reporter system has two uses in the monitoring of osteogenic differentiation. First, it is a less-toxic and transient expression vector of Venus both *in vitro* and *in vivo* in mature osteoblasts of various species^10–12^. Second, it can be used as an efficient site-specific gene-targeting vector through homologous recombination in order to establish reporter knock-in (KI) cell lines^13–16^ without introducing DNA double-strand breaks on and off target sites using programmable nucleases^17^. Using this system, we established a KI human iPSC line to monitor osteogenic differentiation and isolated living mature osteoblasts by fluorescence-activated cell sorting (FACS) from mixed murine POB cultures.

## Results

### Propagation and titration of HDAdVs

**Figure 1:**
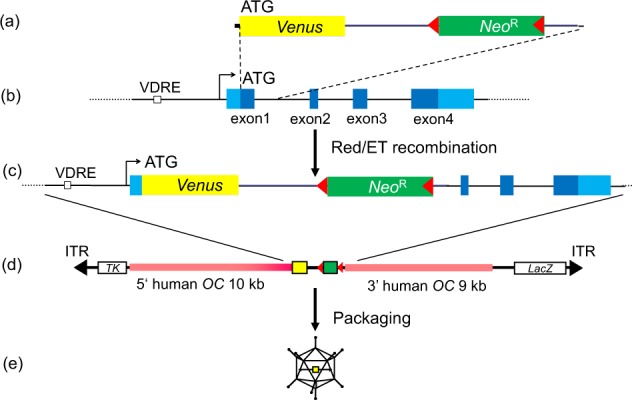
The structure of the HDAd-hOC-Venus vector. (**a**) A 2.6-kb PCR fragment of the Venus-pA-*FRT*-PGK-EM7-neo-pA-*FRT* cassette was amplified using PCR primers with 40-nt homology to the *OC* locus. ATG: translation initiation codon. *Neo*^*R*^: neomycin-resistant gene cassette. Red triangles: *FRT* sites. (**b**) Human *OC* locus. VDRE: vitamin D receptor regulatory element. (**c**) The human *OC* locus with the insertion of the Venus gene by Red/ET homologous recombination. (**d**) Linearized pHDAd-hOC-Venus vector constructed by subcloning a total of 21.4 kb of homologies, including the marker cassette, into pAMHDAdGT8-4. Orange bars: 5′ and 3′ human *OC* gene homology arms. *TK*: herpes simplex virus thymidine kinase gene. *LacZ*: *E*. *coli* β-galactosidase gene. Black triangles: adenoviral inverted terminal repeat (ITR). (**e**) HDAd-hOC-Venus vector packaged as virus.

The structures of HDAd-hOC-Venus and HDAd-CAG-Venus are shown in Fig. 1, Supplementary Fig. S1. HDAd-hOC-Venus encodes the Venus fluorescent reporter gene under the control of human *OC* locus while HDAd-CAG-Venus has a constitutively active strong promoter to drive Venus in almost every cell type. HDAd-hOC-Venus and HDAd-CAG-Venus vector was propagated for 4 rounds in 116 cells; after purification, the physical titer was 9.8 × 10^10^ vector particle (vp)/ml and 8.7 × 10^10^ vp/ml, respectively (determined by quantitative Southern hybridization). The infectious titer of HDAd-hOC-Venus and HDAd-CAG-Venus vector was 3.7 × 10^10^ β-gal-transducing unit (btu)/ml and 3.3 × 10^10^ GFP-transducing unit (gtu)/ml, respectively. The multiplicities of infection (MOIs) of HDAd-hOC-Venus for various cell types were optimized based on the transduction efficiency of HDAd-CAG-Venus. MG-63 or HeLa cells were infected with the HDAdVs at an MOI of 2000 or 100 vp/cell, respectively. At this MOI, 99% of the cells were transduced by the HDAd-CAG-Venus vector (Supplementary Fig. S2). Human induced osteoblasts or mouse POBs, on which bone-like nodules were formed, were infected with the HDAdVs at an MOI of 1,000. With this MOI, 60% of the cells (including both osteoblasts forming bone-like nodules and the, surrounding cells) were transduced with the HDAd-CAG-Venus vector (Supplementary Fig. S3). Then, the promoter-dependent expression from hOC-Venus and its dose-dependency on 1α,25(OH)_2_D_3_ (active vitamin D3; VD3) were examined by FACS and a quantitative reverse transcription polymerase chain reaction (qRT-PCR) in MG-63 cells. The mean fluorescence intensity (MFI) of the total cells (Supplementary Fig. S4a,d) increased after VD3 treatment in a dose-dependent manner, in parallel with the mRNA levels of the *Venus* gene itself (Supplementary Fig. S4b) and of the *OC* gene (Supplementary Fig. S4c) in MG-63 cells.

### The Venus expression in HDAd-hOC-Venus-infected bone-like nodules induced from human osteoblast cultures

To confirm the utility of our HDAdV vector, HDAd-hOC-Venus was used to directly infect human osteoblast cultures induced from the iPSC line SeVdp (KOSM) #7 (hereafter TIG3/KOSM) with the HDAd-hOC-Venus vector at an MOI of 1,000. The specific expression of the hOC-Venus construct at bone-like nodules was observed (Supplementary Fig. S3), which was confirmed by alizarin red staining (Supplementary Fig. S7).

### The Venus gene expression at bone-like nodules induced from OC-Venus knock-in hiPSC lines

**Figure 2:**
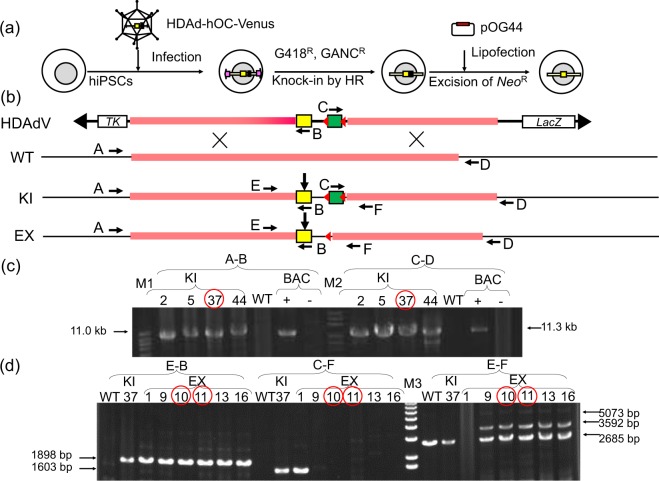
Knock-in and excision of the Venus cassette at the OC locus in hiPSCs. (**a**) A schematic illustration of OC-Venus knock-in with HDAdV and cassette removal by Flp recombinase. (**b**) The structure of the target *OC* locus at each step. HDAdV: HDAd-hOC-Venus vector. WT: wild-type allele. KI: the OC-Venus knock-in allele. EX: the KI allele after excision of the *Neo*^*R*^ cassette. The PCR primers are shown as black arrows with capital letters A to F (corresponding to the list of Table S1b). Yellow box: *Venus* gene, Green box: neomycin-resistant (*Neo*^*R*^) gene cassette. Red triangles: *FRT* sites. (**c**) The results of a long PCR to confirm precise knock-in into the target *OC* locus by homologous recombination. Primer set A-B amplified a left-side 11.0-kb fragment. Primer set C-D amplified a right-side 11.3-kb fragment. BAC (−) is a negative control template of original BAC (RP11-54H19) DNA. BAC (+) is a positive control template of the BAC DNA after cassette insertion (Fig. 1c). KI #37 with red circles was selected for further experiments. M1: Kb DNA Ladder (Agilent Technologies). M2: 1 Kb DNA Extension Ladder (Thermo Fisher Scientific). (**d**) The results of a PCR to confirm precise cassette excision by Flp recombinase. Primer set E-B amplifies a left-side 1898-bp fragment. Primer set C-F amplifies the right-side 1603-bp fragment, which will disappear in EX clones. Primer set E-F amplified a fragment spanning left to right, 2685-bp from the WT allele and 3592-bp from the EX allele. Another 5073-bp fragment from the KI allele should be amplified for KI #37 lane but was not visible in this gel due to the competitive PCR conditions. KI# 37 was a heterozygous knock-in clone. EX #10 and #11 (red circles) were selected for further experiments. M3: 1 Kb Plus DNA Ladder (Thermo Fisher Scientific). Full length gel images of (**c**,**d**) are shown in Supplementary Fig. S5a,b.

To compare the specificities of Venus expression from the HDAd-hOC-Venus vector and the endogenous OC expression, OC-Venus KI human iPSC lines were established by homologous recombination after the infection of the wild-type human iPSC line with the same HDAd-hOC-Venus vector (Fig. 2). After positive selection with G418 and negative selection with ganciclovir (GANC), 6 of 20 of G418/GANC double-resistant colonies were confirmed to be OC-Venus KI clones by a genomic PCR. The results from four representative KI clones are shown (Fig. 2c, Supplementary Fig. S5a). One of the OC-Venus knock-in human iPSC lines, KI #37 (OCVneo37), was transfected with pOG44, an Flp recombinase expression plasmid, to excise the neomycin-resistant gene cassette, because the presence of a drug-resistant gene may interfere with the regulation of the reporter gene expression. Six of 24 single colonies lost resistance to G418 after cloning. Five of them were confirmed to be neo cassette-excision (EX) clones (Fig. 2d, Supplementary Fig. S5b). Two of the neo cassette-excision human iPSC lines, EX #10 (OCV37FF10) and #11 (OCV37FF11), were subjected to the following differentiation assays.

**Figure 3:**
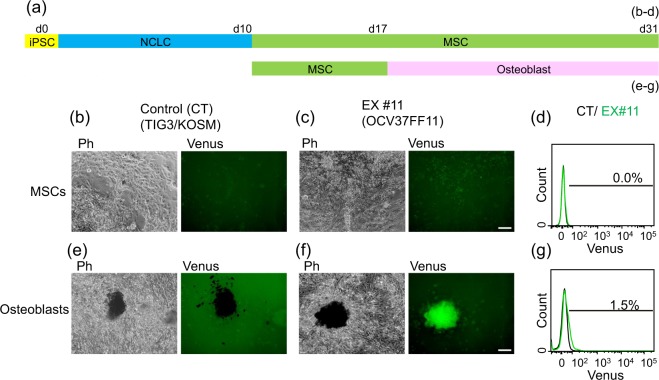
The specific expression of Venus at bone-like nodules in human induced osteoblasts from the OC-Venus knock-in hiPSC line. (**a**) The timeline of human osteoblast induction. Each sampling time is indicated as (**b**–**d**) for MSCs and (**e**–**g**) osteoblasts, respectively. MSCs and osteoblasts induced from control and OC-Venus knock-in human iPSC lines were observed live under an IX81 fluorescence microscope, using a 10x objective lens. Ph: phase contrast images obtained with an exposure time of 10 ms in grayscale. Venus: GFP filter images obtained with an exposure time of 500 ms in green. Scale bar: 100 μm. (**b**) MSCs without osteoblast induction derived from the control (CT; TIG3/KOSM) line. (**c**) MSCs without osteoblast induction from the OC-Venus knock-in (EX #11; OCV37FF11) iPSC line. (**d**) MSCs from (**a**,**b**) were dissociated and analyzed using a FACS Area III. The black line indicates the FACS histogram of CT and the green line indicates that of EX #11. (**e**) Osteoblasts derived from CT on day 31. (**f**) Images of living osteoblasts from EX #11 at day 31. (**g**) Osteoblasts from (**d**,**e**) were dissociated and analyzed using a FACS Area III. The black line indicates the FACS histogram of CT and the green line indicates that of EX #11.

The unmodified control human iPSC line (TIG3/KOSM) and the EX lines (OCV37FF10 and OCV37FF11) were induced into neural crest-like cells (NCLCs) for 10 days, and then into mesenchymal stromal cells (MSCs) for 7 days. Bone-like nodules were formed after the induction of MSCs into the osteoblast lineage for 14 days (Fig. 3a). In cells without osteogenic induction, fluorescence was not detected by fluorescence microscopy (Fig. 3b,c) or FACS (Fig. 3d). The bone-like nodules of the control (CT) lines did not show Venus fluorescence (Fig. 3e), while those of EX lines did (#11; Fig. 3f, #10; Supplementary Fig. S6a). The fluorescence-emitting bone-like nodules were positively stained by alizarin red (Supplementary Fig. S7). The proportion of Venus-positive cells appeared to be no more than 1.5% (Fig. 3g, Supplementary Fig. S6b). Of note, the pattern of Venus expression was similar to that observed in human osteoblast cultures induced from the wild-type iPSC line and infected with HDAd-hOC-Venus, confirming the highly specific expression of HDAd-hOC-Venus.

### The Venus expression in HDAd-hOC-Venus -infected bone-like nodules induced from mouse osteoblast cultures

**Figure 4:**
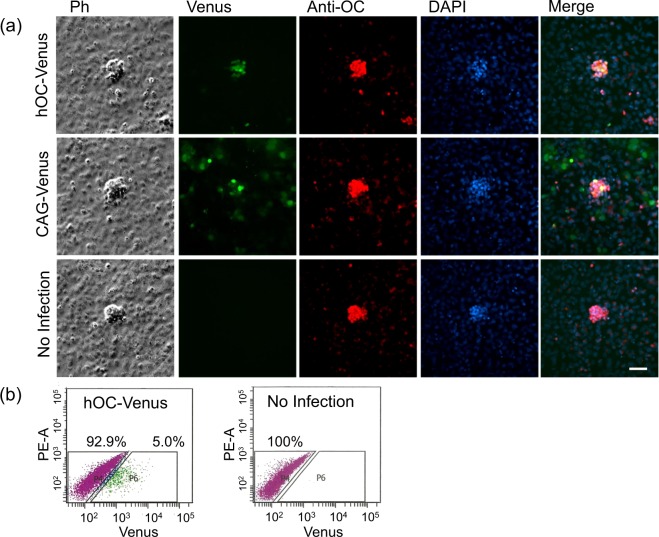
The specific expression of Venus at bone-like nodules in mouse primary osteoblast cultures infected with HDAd-hOC-Venus. (**a**) Mouse POBs were cultured for 6 days and then infected with either HDAd-hOC-Venus or HDAd-CAG-Venus at an MOI of 1,000. Two days later, the cells were stained with an anti-OC antibody. Negative control, uninfected mouse POBs. All images were taken under an IX81 microscope using a 20x objective lens. Ph: phase contrast images obtained with an exposure time of 10 ms in grayscale. Venus: GFP filter images obtained with an exposure time of 500 ms in green. Anti-OC: Immunostaining images of anti-OC detected by Alexa Fluor 594 with an exposure time of 1 s in red. DAPI: DAPI staining images with an exposure time of 1 s in blue. Merge: Merged images of the three colors. Scale bar: 50 μm. (**b**) FACS scatterplots of POBs infected with HDAd-hOC-Venus. The fractions with Venus-positive (+) and Venus-negative (−) cells were isolated by two-color FACS using hOC-Venus (Venus) and autofluorescence (PE-A) as the X- and Y-axes, respectively.

One of the advantages of the HDAdV is that it can also transduce cells from various species other than human. The mature osteoblast-specific expression of hOC-Venus was further examined in cultures of mouse POBs prepared from calvaria. In the cultures of POB, numerous bone-like nodules were formed by day 7. When these cultures were infected with the HDAd-hOC-Venus vector, Venus was specifically expressed in the cells forming bone-like nodules (Fig. 4a). Immunostaining using an anti-OC antibody also specifically labeled the bone-like nodules. The merged images of the HDAd-hOC-Venus expression and the anti-OC staining indicated that the expression of hOC-Venus was colocalized with that of endogenous OC. In contrast to hOC-Venus, CAG-Venus was expressed not only in the nodule-forming cells, but also in the surrounding cells, which were not stained with anti-OC antibody, confirming the specificity of the hOC-Venus expression (Fig. 4a).

### A comparison of the Venus expression controlled by the 19-kb human OC locus and the 3.8-kb human OC promoter

**Figure 5:**
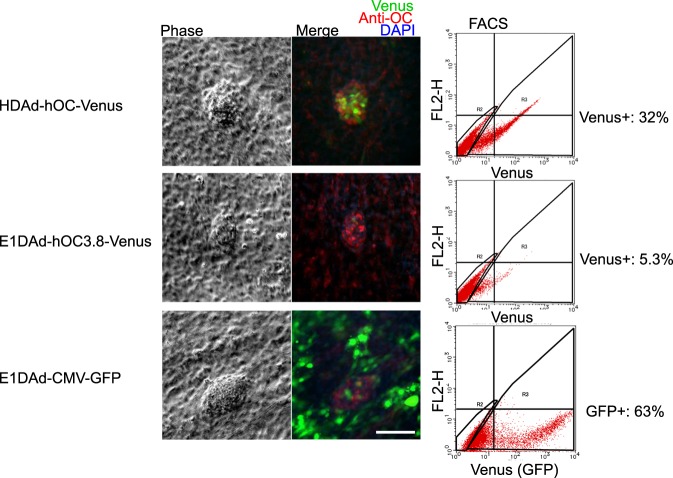
Mouse primary osteoblasts infected with HDAd-hOC-Venus and E1DAd-hOC3.8-Venus. Mouse POBs were cultured for 6 days and then infected with either HDAd-hOC-Venus, E1DAd-hOC3.8-Venus, or E1DAd-CMV-GFP at an MOI of 1,000. Three days later, the cells were stained with an anti-OC antibody, and observed under an IX81 fluorescence microscope using a 20x objective lenses. Ph: phase contrast images obtained with an exposure time of 10 ms in grayscale. Merge: GFP filter image in green, Anti-OC image in red, DAPI image in blue. All of the fluorescence images were obtained with an exposure time of 1 s. Scale bar: 100 μm. FACS: FACS scatterplots of the primary osteoblasts infected with each AdV vector and without immunostaining were created using a FACS Calibur. The X- and Y-axes show hOC-Venus (Venus) and autofluorescence (FL2-H), respectively.

To compare the effects of the 19-kb *OC* locus in HDAd-hOC-Venus and the 3.8-kb *OC* promoter, used in other studies, in driving the reporter gene, HDAd-hOC-Venus, E1DAd-hOC3.8-Venus and E1DAd-CMV-GFP were used to infect mouse POB cultures at the same MOI of 1,000 (Fig. 5). E1DAd-hOC3.8-Venus and E1DAd-CMV-GFP are both E1-deleted AdVs expressing the reporter gene under the control of the 3.8-kb *OC* promoter and the CMV enhancer/promoter, respectively. The transduction efficiency of E1DAd-CMV-GFP was 63% (Fig. 5). While the expression of HDAd-hOC-Venus at the bone-like nodules of mouse POBs was strong and specific (Fig. 5), that of E1DAd-hOC3.8-Venus showed a much weaker signal (Fig. 5). The infectious titers of the two vectors are estimated to be similar or even higher for E1DAd-hOC3.8-Venus, because when they were used to infect HeLa cells at the same MOI of 100, the Venus-transduction efficiency of cells infected by E1DAd-hOC3.8-Venus was almost equal to or higher than that of cells infected by HDAd-hOC-Venus with 1, 10, 100 μM VD3 induction (Supplementary Fig. S8). While the simple VD3-dependent expression was reproduced in HeLa cells by the minimal OC3.8 promoter with VDRE, the OC transcription might be regulated in a more complicated manner in the 19-kb *OC* locus.

### Characterization of hOC-Venus-expressing primary mouse osteoblasts

**Figure 6:**
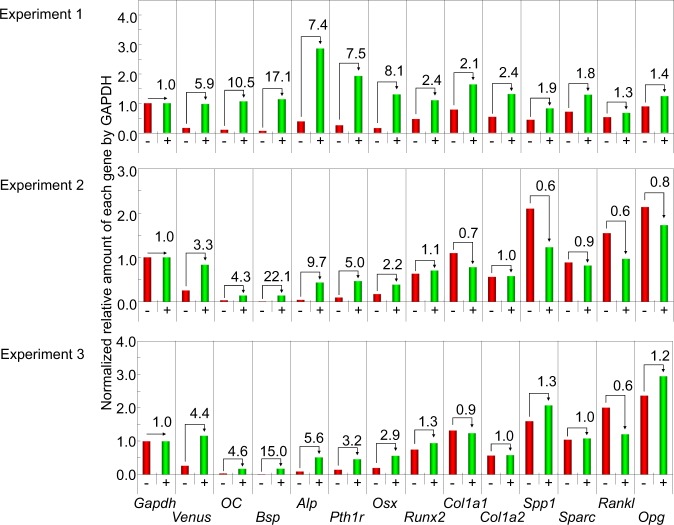
The expression of osteoblast marker genes in hOC-Venus (+) and (−) cells. The normalized relative amounts of osteoblast marker genes in hOC-Venus (+) and (−) POBs isolated by FACS in three experiments (Experiment 1, 2, 3) were analyzed by qRT-PCR. Y-axes represent relative amounts of each gene after normalization by the internal control (*Gapdh*). The values indicated near the arrows connecting the top of the columns represent the ratios of the normalized relative amounts in the hOC-Venus (+) cells to those of the (−) cells in each experiment. The abbreviations of the genes correspond to those shown in the footnote of Table S1.

We then characterized the expression profile of the osteoblast marker mRNAs in the bone nodule-forming osteoblasts. Because it is likely that those cells derived from mouse POBs are more genuine than those induced from hiPSCs, we chose the mouse POB culture system as a source of mRNA. The HDAd-hOC-Venus-infected mouse POB cells were sorted by FACS using the fluorescence intensity of Venus, since no Venus (+) cells were detected in the non-infected cultures (Fig. 4b). As the proportion of Venus (+) cells fluctuated from 5% (Fig. 4b) to 30% (Fig. 5) between the experiments, we repeated the experiments independently three times. The Venus (+) fraction in the HDAd-hOC-Venus infected cultures was 5.0% of the total cells (Fig. 4b), and the purity after FACS sorting was 87% (Supplementary Fig. S9) in a representative experiment. The relative expression levels of mRNAs expressed in osteoblasts in the hOC-Venus (−) and hOC-Venus (+) cells were examined by a qRT-PCR. Regardless of some inconsistent results between experiments, reproducible patterns were observed (Fig. 6). Among the twelve genes examined, the expression levels of five endogenous genes (*OC* [4.3–10.5 fold], *Bsp* [15.0–22.1 fold], *Pth1r* [3.2–7.5 fold], *Alp* [5.6–9.7 fold] and *Osx* [2.2–8.1 fold]) in the Venus (+) cells were higher than those in the Venus (−) cells (Fig. 6). The expression of an osteogenic transcription factor, *Runx2*, was also slightly higher (1.1–2.4 fold) in hOC-Venus (+). In contrast, the expression levels of four genes of the secretory proteins (*Col1a1*, *Col1a2*, *Spp1* and *Sparc*) and two genes related to bone resorption (*Opg* and *Rankl*) did not differ to a statistically significant extent between Venus (+) and Venus (−) cells.

## Discussion

In the present study, we showed the dual usage of a novel adenoviral vector, HDAd-hOC-Venus, which allows for both the generation of knock-in reporter cell lines by homologous recombination in human iPSCs and postnatal visualization and separation of bone-forming mature osteoblasts by transient infection into mouse and human cells.

Our vector system has distinct advantages over other methods for tracing osteoblasts. First, our HDAdV-based system allows for the transient expression of Venus without chromosomal integration in the target cells. Thus, unlike transgenic animals or lentivirus-based systems, the vector DNA will be eventually lost from the infected cells after several cell divisions. This feature is potentially quite advantageous for the clinical application of the vector, such as for the enrichment of mature osteoblasts for transplantation. Second, adenovirus-mediated gene transfer is more efficient than other viral and non-viral methods in various cell types. In contrast to the GFP transgenic mice driven by the human *OC* promoter^4^, our HDAdV system is more versatile and can be used to visualize the OC-positive cells in unmanipulated animal species, including humans. Born *et al*. used nucleofection to deliver the 3.8-kb human OC promoter-GFP construct into human bone marrow-derived cells with a maximum transfection efficiency of 25%^5^; thus, a double-gene construct of the OC promoter-GFP and CMV promoter-H2B-RFP had to be used to enrich transfected cells by sorting before their differentiation experiment^6^. However, the transduction efficiency of the double-gene construct was even lower (5.3–5.6%), probably because of the larger size of the construct, as discussed by the authors. Compared with nucleofection, the transduction efficiencies of adenoviral vectors of approximately 30 kb into mouse and human osteoblasts were as high as 60% at an MOI of 1,000, which was used in this study (Fig. 5, Supplementary Fig. S2). No reduction in the cell viability was observed around an MOI of 1,000, and we have observed no negative consequences due to adenoviral infection below an MOI of 10,000. Third, while antibody-based cell sorting is useful for proteins expressed on the cell surface, the reporter system with the promoter-specific expression of fluorescent protein allows us to sort target cells expressing proteins for which no suitable antibody is available as well as intracellular or secreted proteins. Although a previous study demonstrated that using OC antibodies for FACS is useful for detecting circulating osteoblasts^18^, the transient expression of Venus by OC-positive cells during induction into an osteogenic lineage allows us to evaluate the degree of differentiation via microscopy and conduct continuous experiments without fixing the cells for antibody staining. This is an important advantage, especially for bone research, which involves time-consuming and complicated induction processes. Fourth, the tissue-specific expression achieved using the HDAdV vector is superior to that achieved when using the first-generation adenoviral vector due to its high capacity to accept long regulatory elements of up to 20 kb and minimal viral enhancers, which will interfere with the expression of reporter genes^19^. In support of this, when POB forming bone-like nodules were infected with HDAd-hOC-Venus, Venus was specifically expressed in the bone-like nodules, which were stained with anti-OC antibody (Fig. 4a). In addition, the expression levels in the bone-like nodules that were achieved using the 19-kb human *OC* locus were higher than those achieved using the 3.8-kb OC promoter (Fig. 5). These results indicate that, to precisely reproduce tissue-specific expression of an endogenous gene, it is critical to use a large genomic locus to drive a reporter gene. HDAdVs have a cloning capacity of roughly 30 kb and are therefore an ideal vector for this purpose. Finally, using the same HDAdV, we were able to establish knock-in human iPS cell lines with which we can visualize the developmental expression patterns of the OC gene. Although the targeting efficiency when using infection with HDAdVs is reportedly lower than when using artificial nucleases^17^, the former approach is roughly 300-fold as efficient as that by traditional electroporation of naked plasmid DNA^13^. In addition, this vector system can obviously be used in combination with artificial nucleases to achieve even more efficient gene targeting^20^. Using high-capacity HDAdVs has additional advantages over traditional methods, including efficient transduction into a wide range of cell types, efficient knock-in of large DNA cassettes, simultaneous introduction of multiple modifications to a large DNA region, and no risk of off-target cleavage caused by artificial nucleases, as discussed previously^13,14,17^.

However, despite these advantages, one limitation associated with the HDAd-hOC-Venus vector might be that the infection efficiency could not reach 100% at an MOI of 1,000 in our experiment, even though this infection efficiency was superior to that of nucleofection. Thus, the hOC-Venus (−) fraction could contain endogenous OC-positive cells that were not infected. Infection at higher MOIs, at 10,000, resulted in significant cell death of POBs. To further improve our system, a higher infection efficiency without cytotoxicity - possibly through the use of adenoviral vectors from other serotypes or a method that excludes uninfected cells - is therefore required. Furthermore, propagating HDAdVs takes more time than traditional gene targeting methods using plasmid DNA and requires a biohazard level 2 facility.

In the present study, we successfully enriched OC-expressing mature osteoblasts from the mixed POB cultures using the HDAd-hOC-Venus virus. We found that OC-expressing mature osteoblasts co-expressed *Bsp*, *Pth1r*, *Alp* and *Osx* mRNAs at higher levels than in immature osteoblasts. The results are expected for *Bsp* because it has been reported to be highly expressed in mature mineralizing osteoblasts^21^, while *Alp* and *Pth1r* have been regarded as an early marker of osteogenesis. The expression of *Alp* and *Pth1r* might start at an early stage and increase during maturation, as reported in some *in vitro* analyses using the MC3T3-E1 cell line^22^. In addition, our results suggested that Osx is involved in the later stages of osteogenesis and that it regulates the expression of *OC*, *Bsp*, *Pthr1* and *Alp* in mature osteoblast forming bone-like nodules *in vitro*. Further analyses of the binding sequences of Osx in the promoter/enhancer regions in those genes may therefore help us to understand the molecular mechanisms underlying such differentiation-dependent transcription of the genes related to bone formation.

In summary, we established a novel HDAdV-based system to detect living mature osteoblasts using the long regulatory sequences of the human *OC* gene. Our HDAdV-based hOC-Venus reporter will therefore be useful to visualize, isolate and characterize mature osteoblasts in various systems. Such HDAdV-based transient reporter systems would also be widely applicable to isolate other cell lineages or tissues from various species, including humans.

## Methods

### Cell culture

The 116, a Cre recombinase expressing HEK293 cell line (kindly provided by Dr. Phillip Ng)^23^, was cultured in MEM (Nacalai tesque, Japan) with 10% fetal calf serum (FCS; MP Biomedicals, Solon, OH). The human osteosarcoma cell line, MG-63 (Cell Resource Center for Biomedical Research Institute of Development, Aging and Cancer Tohoku University)^24^ was cultured in MEM with 10% FCS supplemented with 1 mM sodium pyruvate (Sigma-Aldrich, St. Louis, MO) and 1x non-essential amino acids (Sigma-Aldrich). The human cervical carcinoma cell line, HeLa, and HEK293 cell line were cultured in DMEM (Nacalai tesque) with 10% FCS. The human iPSC line, TIG3/KOSM (formerly termed SeVdp (KOSM) #7)^25^ was maintained as on-feeder^26^ or feeder-free^27^ culture, as described previously.

### Construction and preparation of HDAdVs

To generate the human OC-Venus HDAdV vector (HDAd-hOC-Venus), RP11-54H19, a BAC clone containing the human *OC* locus (BACPAC resources, Children’s Hospital & Research Center at Oakland, Oakland, CA), was modified using the Red/ET recombination technique^28^. An *FRT*-PGK-EM7-neo-pA-*FRT* cassette was inserted into a single *Not*I (New England Biolabs, Ipswich, MA) site of pCS2-Venus (kindly provided by Dr. Atsushi Miyawaki)^9^. Next, the Venus-pA-*FRT*-PGK-EM7-neo-pA-*FRT* cassette was amplified by a PCR using primers with a 40-nt homology sequence to the target site (Table S1a upper) and inserted into exon 1 of the *OC* gene on the BAC (Fig. 1a–c). The ATG start codon of Venus was fused in-frame with the ATG of the *OC* gene. Subsequently, a total of 21.4-kb *OC* gene locus, including the marker cassette, was retrieved into a PCR-amplified pBR322 vector backbone with a second primer set (Table S1a lower). The entire cassette was excised by *Sal*I (New England Biolabs) and inserted into an HDAdV plasmid, pAMHDAdGT8-4^14^. The resultant pAMHDAdGT-hOC-Venus plasmid was linearized by *Pme*I (New England Biolabs) (Fig. 1d) and packaged into virus particles (Fig. 1e) by transfection into 116 cells with the addition of AdNG163R-2 helper virus (kindly provided by Dr. Phillip Ng). The viral vector was propagated by serial passages in the 116 cell line with AdNG163R-2 and purified, as described previously^23,29,30^. The physical titer of the vector was determined as the copy number of viral genomic DNA by a quantitative Southern analysis^30^. The infectious titer was determined as β-gal-transducing units (btu) by X-gal staining on the 293LP cell line^29^. HDAd-CAG-Venus (formerly termed HDAdVenus-geo-TK^13^) was also propagated and used as a constitutive Venus-expressing control (Supplementary Fig. S1). The infectious titer of this vector was determined as GFP(Venus)-transducing units (gtu) measured on 293A cells by a FACS analysis. The MOI for each cell type was defined as the vector copy number to the cell number.

### Generation of knock-in reporter hiPSC lines with HDAd-hOC-Venus vector

A control hiPSC line, TIG3/KOSM, maintained on SNL feeder cells with hES medium was treated with CTK solution and suspended in hES medium as small clumps, as previously described^26^. They were then infected with HDAd-hOC-Venus at an MOI of 300 and plated onto SNL feeder cells. G418 selection (50 μg/ml; Nacalai tesque) was started 2 days after infection. After 3 weeks, the surviving colonies were transferred to 96-well plates and ganciclovir (GANC) selection (2 μM; Thermo Fisher Scientific, Waltham, MA) was started. G418/GANC double-resistant clones were characterized by a genomic PCR using the primers shown in Table S1b and Fig. 2 with PrimeSTAR GXL polymerase (Takara Bio, Japan), in accordance with the manufacturer’s instructions. The resulting OC-Venus KI hiPSC lines were habituated to feeder-free culture using StemFit AK03 medium (Ajinomoto, Japan) and iMatrix-511 (Nippi, Japan) for two passages, as previously described^27^. They were then transfected with pOG44 (Thermo Fisher Scientific) using TransIT®-LT1 Transfection Reagent (Takara Bio), according to the manufacturer’s protocol, and plated onto iMatrix-511-coated 6-cm culture dishes. Single colonies were picked-up and the resistance to G418 was checked on duplicated plates. The clones that were sensitive to G418 were selected as candidate clones with neo cassette-excision (EX) by Flp recombinase, and the excision of the neo cassette was confirmed by a PCR using the primers shown in Table S1b and Fig. 2.

### Differentiation of human iPSCs into osteoblasts

The control hiPS cell line, TIG3/KOSM, and OC-Venus KI hiPS cell lines were cultured under Matrigel-coated feeder-free conditions with mTeSR-1 (BD Bioscience, San Jose, CA). NCLC induction was performed for 10 days, as previously described^31^. Induced NCLCs were maintained with medium containing DMEM (Sigma-Aldrich), 10% FBS (Thermo Fisher Scientific), 1% L-glutamine (Nacalai Tesque) for an additional 7 days. After additional culturing, the cells showed vigorous expansion and appeared to be MSCs. The MSCs were induced into osteoblasts with Osteogenic Differentiation Medium BulletKit™ (Lonza, Switzerland) on a 6-well dish or an 8-well glass chamber slide II (AGC Techno Glass, Japan) for 14 days (Fig. 3a).

### Primary osteoblast isolation and the nodule formation assay

Mouse POBs were isolated according to the published methods^2,32^. POBs were obtained from the calvariae of neonatal C57BL/6J Jc1 mice (Clea Japan, Japan). A mixture of 0.1% collagenase (FUJIFILM Wako, Japan) and 0.2% dispase was used to dissociate cells from the bone fragments. POBs were then seeded onto either a 6-well plate or an 8-well chamber slide II at a density of 2.0 × 10^6^ or 3.2 × 10^5^ cells/well, respectively. The cells were cultured in αMEM (Thermo Fisher Scientific) with 10% FCS supplemented with 50 U/ml streptomycin (Thermo Fisher Scientific) and 50 μg/ml penicillin (Thermo Fisher Scientific). The medium was changed every 48 h for 1~2 weeks until bone-like nodules were formed.

### Immunohistochemistry and microscopy

The human osteoblasts induced from the control iPSC line or mouse POBs infected with the HDAdVs were immunostained using a standard method with an anti-OC rabbit polyclonal antibody, LSL-LB-4005 (Cosmo Bio, Japan) as a primary antibody and Alexa Fluor 594-conjugated anti-rabbit IgG antibody (Thermo Fisher Scientific) as a secondary antibody. The cells were mounted with ProLong Gold antifade reagent with 4′,6-diamidino-2-phenylindole (DAPI) (Thermo Fisher Scientific), and were then examined under a fluorescence microscope, IX81 (Olympus, Japan), with a CCD camera, CoolSNAP HQ (Photometrics, UK) or ORCA-ER (Hamamatsu photonics, Japan).

### The FACS analysis and flow cell sorting

A FACS analysis of cells infected with the HDAdV vectors or differentiated human OC-Venus KI cells was performed using a FACS Calibur (BD Biosciences) or the FACS Aria III (BD Biosciences). FACS isolation according to the expression of Venus was also performed using a FACS Aria II (BD Biosciences). Before FACS, the cells were counter-stained with propidium iodide (PI) to distinguish dead and living cells. The FlowJo software program (BD Biosciences) was used for the data analyses.

### Quantitative RT-PCR

Total RNA was isolated from the cells using an RNeasy Mini Kit (Qiagen, Germany) and then reverse-transcribed into cDNA using a PrimeScript 1st strand cDNA Synthesis Kit (Takara Bio)^9,33^. In addition to the reporter gene, *Venus*, the following osteoblast marker genes were analyzed: secretory proteins (*OC*, *Bsp*, *Col1a1*, *Col1a2 Spp1* and *Sparc*); hormone receptor and enzyme (*Pth1r* and *Alp*); transcription factors (*Osx* and *Runx2*); and proteins related to osteoclast induction (*Opg* and *Rankl*). The specific qRT-PCR primers for each gene are listed in Table S1c. The primers for Venus, human *OC* and glyceraldehyde 3-phosphate dehydrogenase (*GAPDH*), mouse *Osx* and *OC* were designed with the aid of the Oligo 7 software program (Molecular Biology Insights, Cascade, CO). The primers for other genes were designed using the Perfect Real Time support system (Takara Bio). A real-time PCR was performed with SYBR Green PCR Master Mix (Thermo Fisher Scientific) and an ABI PRISM 7000 sequence detection system (Thermo Fisher Scientific). Quantification of the relative amount of mRNA from the Ct value was performed by the relative standard curve method, which is described in the supplier’s instructions (Thermo Fisher Scientific). Human *GAPDH* and mouse *Gapdh* were used as internal controls to normalize the relative amount. The analyses were performed in triplicate for each experiment (n = 3).

### Ethical approval and informed consent

All of the mouse experiments were approved by the animal research committee of Saitama Medical University. All methods were performed in accordance with the relevant guidelines and regulations.

## Supplementary information

Supplementary Methods, Supplementary Figure Legends 1-9, Supplementary Table 1
