# Supplementary material for: Dual usage of a stage-specific fluorescent reporter system based on a helper-dependent adenoviral vector to visualize osteogenic differentiation
Source: Sci Rep. 2019 Jul 4;9:9705. doi: 10.1038/s41598-019-46105-y (PMC6609771; doi:10.1038/s41598-019-46105-y)

Dual usage of a stage-specific fluorescent reporter system based on a helper-dependent adenoviral vector to visualize osteogenic differentiation

Takefumi Sone, Masashi Shin, Takehito Ouchi, Hiroki Sasanuma, Arei Miyamoto, Satoshi Ohte, Sho Tsukamoto, Mahito Nakanishi, Hideyuki Okano, Takenobu Katagiri, Kohnosuke Mitani

Supplementary Methods, Supplementary Figure Legends 1-9, Supplementary Table 1

Supplementary Method

Adenoviral infection of MG-63 and HeLa cells

Viral infection was carried out according to a published method[1]. An optimal MOI was determined for each cell type by using the control HDAd-CAG-Venus vector. For infection with HDAd-hOC-Venus, $1\alpha,25(OH)_2D_3$ (Sigma-Aldrich, St. Louis, MO) was added to the media at a final concentration of 0, 1, 10 and 100 μM to confirm the dose-dependency of the expression of the Venus gene on active vitamin D3 (VD3). The images of live cells were taken under a fluorescence microscope, IX81 (Olympus, Japan), with a CCD camera, CoolSNAP HQ (Photometrics, UK) at 3 days after infection.

Alizarin red staining and microscopy

Alizarin red S staining of human induced osteoblasts from control or OC-Venus KI iPSCs was used to confirm the mineralization of the matrices of induced cells. In brief, the osteogenically induced cells were fixed with 10% paraformaldehyde (PFA) and then stained with Alizarin red S (FUJIFILM Wako, Japan). The images of alizarin red staining were taken using a BZ-X800 (a fluorescence and bright field microscope; Keyence, Japan).

The construction and preparation of first generation AdVs

To compare the 19-kb *OC* gene locus and the 3.8-kb *OC* promoter in driving a reporter gene, a first-generation AdV plasmid with 3.8-kb *OC* promoter was constructed using the AdEasy Adenoviral Vector System (Agilent Technologies, Santa Clara, CA)[2]. The expression cassette with the 3.8-kb OC promoter and Venus gene (4.7 kb in total) was excised from the pAMHDAdGT-hOC-Venus plasmid vector by *Hin*dIII (New England Biolabs, Ipswich, MA) and *Not*I (New England Biolabs) digestion (Supplementary Fig. S1a) and inserted into the pShuttle vector in the reverse orientation to E1 genes, in order to avoid the effects of E1 transcription. The resultant plasmid was linearized by *Pme*I (New England Biolabs) and co-transformed into *E. coli* strain BJ5183 with the adenoviral backbone plasmid, pAdEasy-1. Recombinant adenoviral plasmids were selected on kanamycin and confirmed by restriction digestion. The recombinant adenoviral plasmid, pAdEasyShuttle-hOC3.8-Venus, was then digested with *Pac*I (New England Biolabs) and transfected into 293A cells where it was packaged into virus particles (Supplementary Fig. S1c). The physical titer of the resultant vector, E1DAd-hOC3.8-Venus, was determined as the copy number of viral genomic DNA by a quantitative Southern analysis[3]. A control first-generation AdV, E1DAd-CMV-EGFP, was also prepared in accordance with the manufacturer’s instruction (Supplementary Fig. S1d).

Supplementary Figure Legends

Supplementary Fig. S1. Structure of AdVs

HDAd-hOC-Venus: the *Hin*dIII and *Not*I sites and the hOC 3.8-kb promoter region are indicated. Black triangles: adenoviral inverted terminal repeat (ITR). The more detailed structure of the vector is shown in Fig. 1. HDAd-CAG-Venus: formerly termed HDAdVenus-geo-TK[1]. cHPRT: sequences of the cynomologous monkey hypoxhanthine phosphoribosyltransferase (HPRT) gene locus, inserted as a stuffer. Blue lines: E1/E3-deleted viral backbone of adenovirus serotype 5 (Ad5 [DE1/3]).

Supplementary Fig. S2. The transduction efficiencies of Venus in MG-63 and HeLa cells infected with HDAd-CAG-Venus

MG-63 or HeLa cells were infected with HDAd-CAG-Venus at an optimal MOI of 2000 or 100 vp/cell, respectively. They were observed under an IX81 fluorescence microscope, using a 10x objective lenses 3 days after infection. Ph: phase contrast images obtained with an exposure time of 100 ms in grayscale. Venus: a GFP filter image obtained with an exposure time of 1 s in green. Scale bar: 100 μm.

Supplementary Fig. S3. The bone-like nodule-specific expression of Venus in human induced osteoblasts infected with HDAd-hOC-Venus

Osteoblasts induced from control and OC-Venus knock-in human iPSC lines at day 32 were either infected with HDAd-hOC-Venus or HDAd-CAG-Venus at an MOI of 1000. They were observed under an IX81 fluorescence microscope, using a 10x objective lenses on day 35. Ph: phase contrast images obtained with an exposure time of 10 ms in grayscale. Venus: a GFP filter image obtained with an exposure time of 500 ms in green. Scale bar: 100 μm.

Supplementary Fig. S4. The VD3-dependent expression of Venus in MG-63 cells infected with HDAd-hOC-Venus

MG-63 was infected with HDAd-hOC-Venus and cultured in the medium with increasing concentrations of VD3. Uninfected cells were used as a negative control. ***(a)*** The mean fluorescence intensities were determined by a FACS analysis. ***(b)*** The normalized relative amounts of the *Venus* gene were analyzed by a qRT-PCR. The Y-axis represents the relative amounts normalized by the internal control (*Gapdh*). The analyses were performed in triplicate for each experiment (n=3); the average result is shown with a standard error bar. ***(c)*** The normalized relative amounts of the *OC* gene were analyzed by a qRT-PCR. Y-axis

represents the relative amounts normalized by the internal control. ***(d)*** Fluorescence images of Venus (green) were taken under a fluorescence microscope with a 20x objective lens and an exposure time of 1 s. Scale bar: 100 μm.

Supplementary Fig. S5. Uncropped gel image of PCR fragments shown in Fig. 2

(a) A full-length gel image of PCR fragments amplified from KI clones shown in Fig. 2c. ***(b)*** A full-length gel image of PCR fragments amplified from EX clones shown in Fig. 2d.

Supplementary Fig. S6. The specific expression of Venus at bone-like nodules in human induced osteoblasts from the OC-Venus knock-in hiPSC line, *EX #10*

(a) Images of living osteoblasts from EX #10 at day 31. Indications correspond to those of Fig.3. ***(b)*** Osteoblasts from (a) were dissociated and analyzed using a FACS Area III. The black line indicates the FACS histogram of CT and the green line indicates that of EX #10.

Supplementary Fig. S7. Bone-like nodules expressing Venus stained by alizarin red

Human osteoblasts induced from control and OC-Venus knock-in human iPSC lines on day 31 were stained by alizarin red and bright and fluorescent fields were observed. All of the

images were obtained using a BZ-X800 microscope, with a 4x objective lenses. Alizarin red: bright field images obtained with an exposure time of 2.5 ms. Venus: GFP filter images obtained with an exposure time of 100 ms in green.

Supplementary Fig. S8. The VD3-dependency of Venus-transduction efficiency in HeLa cells infected with HDAd-hOC-Venus and E1DAd-hOC3.8-Venus.

HeLa cells were infected with either HDAd-hOC-Venus or E1DAd-hOC3.8-Venus at an MOI of 100. They were cultured for 1 day at a final concentration of 0, 1, 10 and 100 μM of VD3. Then, the Venus transduction efficiency was measured using a FACS Calibur.

Supplementary Fig. S9. FACS scatterplots of POBs infected with HDAd-hOC-Venus and the purity of isolated cells in Fig. 4b.

hOC-Venus: FACS scatterplots of mouse POBs infected with HDAd-hOC-Venus. The fractions with Venus-positive (+) and Venus-negative (-) cells were isolated by two-color FACS using Venus (Venus) and autofluorescence (PE-A) as the X- and the Y-axes, respectively. Venus (+) and Venus (-): Purity of the isolated cells were analyzed by FACS for Venus (+) and Venus (-) cells.

References

1. Suzuki, K. et al. Highly efficient transient gene expression and gene targeting in primate embryonic stem cells with helper-dependent adenoviral vectors. *Proc Natl Acad Sci U S A* **105**, 13781-13786 (2008).

2. He, T.C. et al. A simplified system for generating recombinant adenoviruses. *Proc Natl Acad Sci U S A* **95**, 2509-2514 (1998).

3. Palmer, D. & Ng, P. Improved system for helper-dependent adenoviral vector production. *Mol Ther* **8**, 846-852 (2003).

Supplementary Table

Table S1. Primer sequences

(a) Primers for Red/ET cloning

Target	Primer	Sequence
Venus-PGKEM7neo / human *BGLAP* locus	forward	CAGCTGAGTCCTGAGCAGCAGCCCAGCGCAGCCACCGAGACACCATGGTGAGCAAGGGCGAGGAGCTGTTCA
	reverse	AGGGCAGAGCTGGGGCAAATGGATTGAGCCTGCAACAAGGTGGTTAGATATCTTTTCGAATTACCCTGTTATCCCTAGGC
pBR322 / human *BGLAP* locus	forward	TGGATGCAAATCTCTCATCTAAGCACGAGTCACCGAGGATGGTTCAAGTCGACAGTAGTAGGTTGAGGCCGTTGA
	reverse	GCCCTGGAAGCTGGCCCTGGAGACATATGGGGAGCAGGCAAAGGTGGTCGACGCCCTGCACCATTATGTTCC

(b) Primers for confirmation of KI

Target	Primer	Sequence
5' arm PCR	A. 5'hBGLAP9615-long-Fw	GATGTCACATGCCCTGTCCTAATCCAAGTCCC
	B. Venus-long-Rv	GCAGGACCATGTGATCGCGCTTCTCGTT
3' arm PCR	C. neo-long-Fw	ATGGCCGCTTTTCTGGATTCATCGACTGTG
	D. 3'hBGLAP31021-long-Rv	TCCGCCTCTCCCAGATGACCCTCCTTACTACTCA
5'3' arm PCR	E. 5'hBGLAP18686-Fw	CTCAGTCTCCCTCTGCCCACCTTGTCACC
	F. 3'hBGLAP23758	CCCATCCCCACAAACCAGGTAATGCCAGT

	-Rv	

(c) Primers for qRT-PCR

Target	Primer	Sequence
Venus cDNA	Venus_GFP F	TGCTGCTGCCCGACAACCACT
	Venus_GFP R	GCGGCGGTCACGAACTCCA
hGAPDH[a] cDNA	RT-GAPDH-Fw2	GAAGGTGAAGGTCGGAGTCA
	RT-GAPDH-Rv2	GCCCCACTTGATTTTGGAG
hOC cDNA	hBGLAP-f2	CCTCACACTCCTCGCCCTA
	hBGLAP-r2	CTTGGACACAAAGGCTGCAC
mGAPDH[b] cDNA	Gapdh F	CTCCACTCACGGCAAATTCAAC
	Gapdh R	CGCTCCTGGAAGATGGTGATG
mOC cDNA	mOG1-f2	CATCTTTCTGCTCACTCTGC
	mOG1-r2	CTTGGACATGAAGGCTTTGTC
mOsx cDNA	Osx F	CCTCTCGACCCGACTGCAGATC
	Osx R	AGCTGCAAGTCTCTGTAACCATGAC
mCol1a1 cDNA	Col1a1 F	CAGGGTATTGCTGGACAACGTG
	Col1a1 R	GGACCTTGTTTGCCAGGTTCA
mCol1a2 cDNA	Col1a2 F	CCAACAAGCATGTCTGGTTAGGAG
	Col1a2 R	GCAATGCTGTTCTTGCAGTGGTA
mRunx2 cDNA	Runx2 F	GCACAAACATGGCCAGATTCA
	Runx2 R	AAGCCATGGTGCCCGTTAG
mAlp cDNA	Alp F	ACACCTTGACTGTGGTTACTGCTGA
	Alp R	CCTTGTAGCCAGGCCCGTTA
mPth1r cDNA	Pth1r F	CTGTGGCAGATCCAGATGCACTA
	Pth1r R	GAAGTCCAATGCCAGTGTCCAG
mBsp cDNA	Bsp F	CAGCCTCGTGGCGACACTTA
	Bsp R	ATTCTGACCCTCGTAGCCTTCATA
mSpp1 cDNA	Spp1 F	TACGACCATGAGATTGGCAGTGA
	Spp1 R	TATAGGATCTGGGTGCAGGCTGTAA
mSparc cDNA	Sparc F	GCATGCGTGACTGGCTCAA
	Sparc R	AAGTCTCGGGCCAACAGCTC
mOpg cDNA	Opg F	CAATGGCTGGCTTGGTTTCATAG
	Opg R	CTGAACCAGACATGACAGCTGGA

mRankl cDNA	Rankl F	CATGTGCCACTGAGAACCTTGAA
	Rankl R	CAGGTCCCAGCGCAATGTAAC

[a] Target's name with "h" at initial indicates human gene.[b] Target's name with "m" at initial indicates mouse gene.

Abbreviations of genes. BGLAP: bone gamma-carboxyglutamic acid-containing protein (also known as osteocalcin), GFP: green fluorescent protein, GAPDH: glyceraldehyde 3-phosphate dehydrogenase, OC: osteocalcin (also known as BGLAP), Osx: osterix, Col1a1: collagen type I a1 chain, Col1a2: collagen type I a2 chain, Runx2: runt-related transcription factor 2, Alp: alkaline phosphatase, Pth1r: parathyroid hormone receptor, Bsp: bone sialoprotein, Spp1: secreted phosphoprotein 1 (also known as osteopontin), Sparc: secreted protein acidic and rich in cysteine (also known as osteonectin), Opg: osteoprotegerin, Rankl: receptor activator of nuclear factor kappa-B ligand.

1

Supplementary Fig. S1

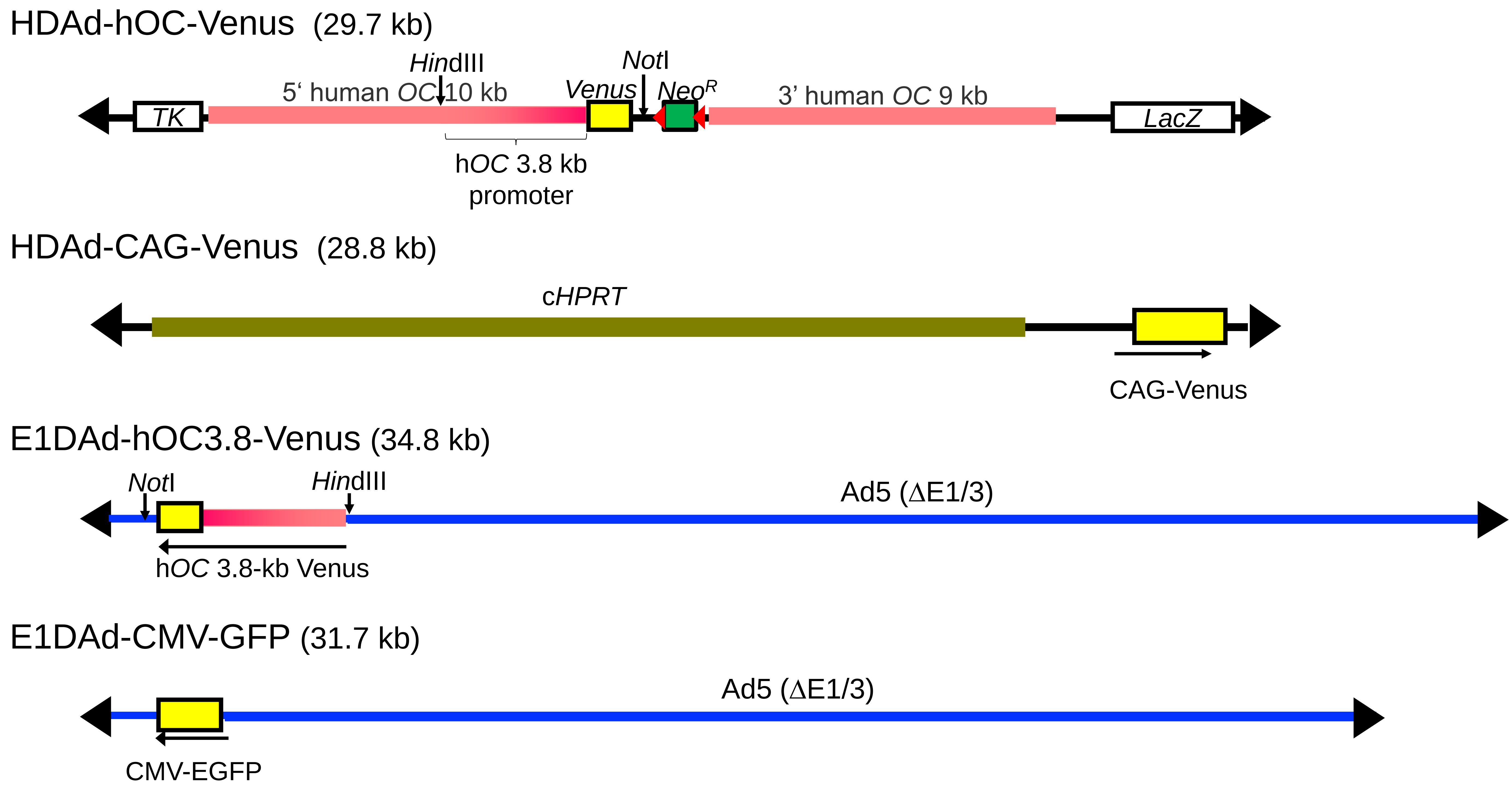

Supplementary Fig. S2

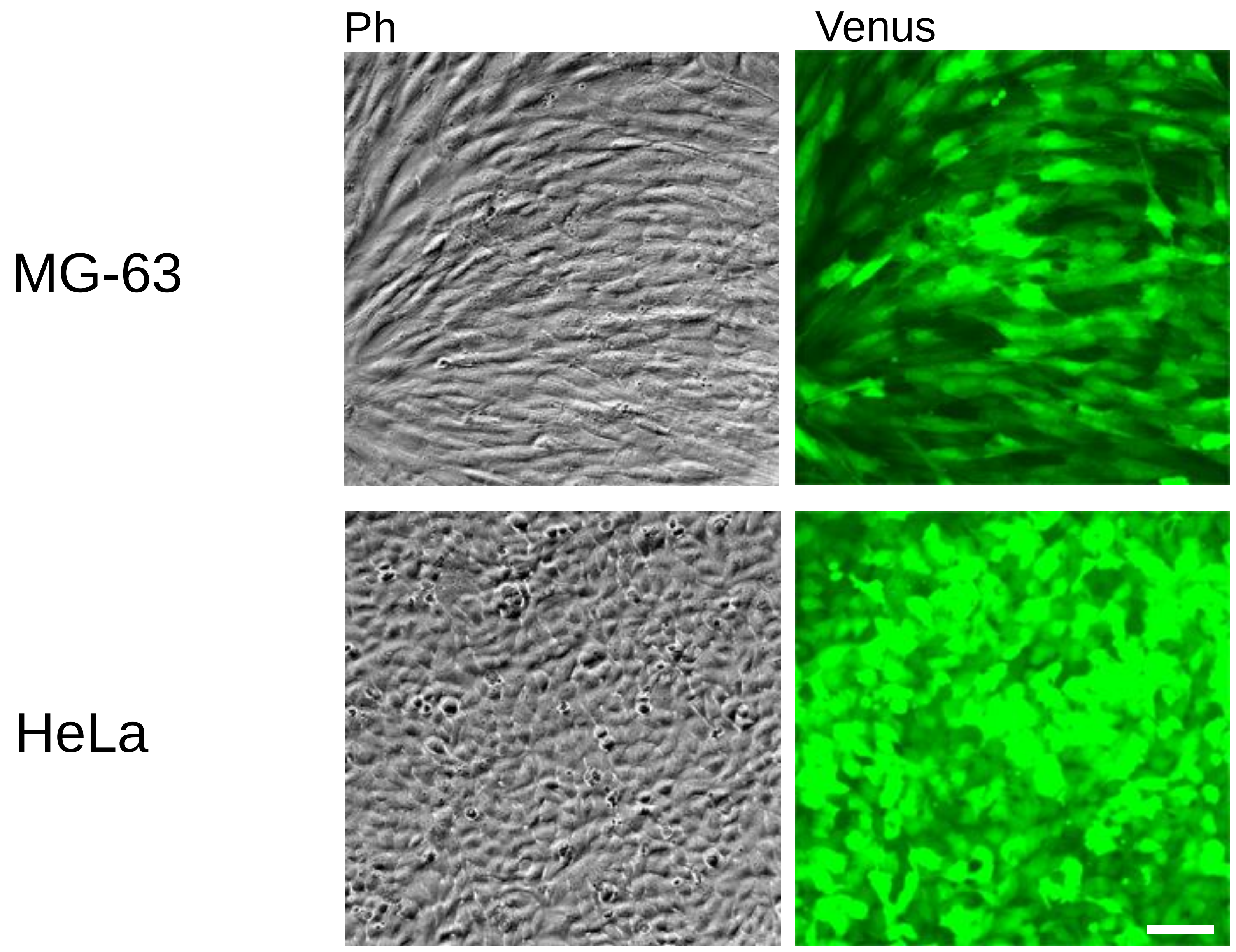

Supplementary Fig. S3

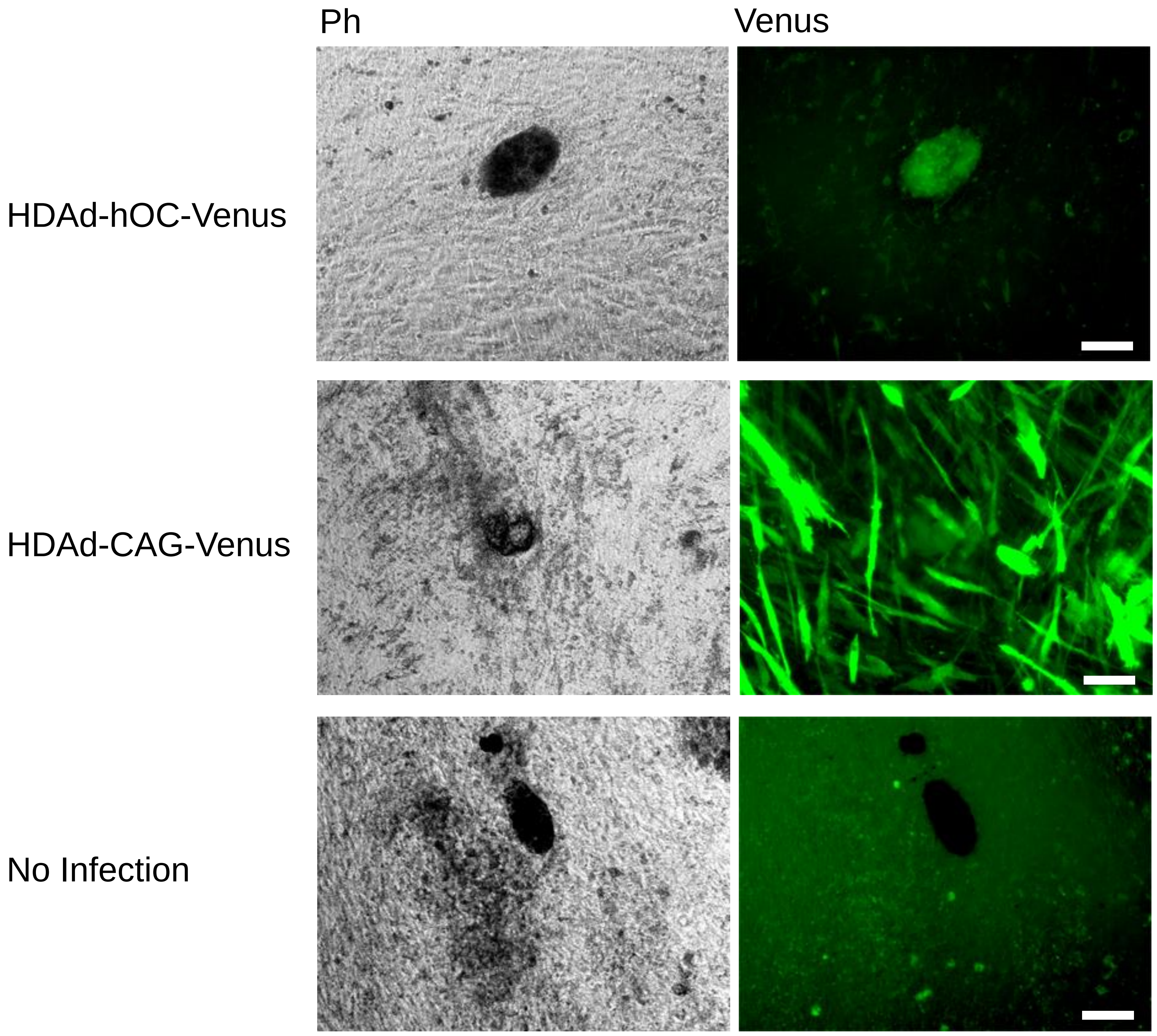

Supplementary Fig. S4

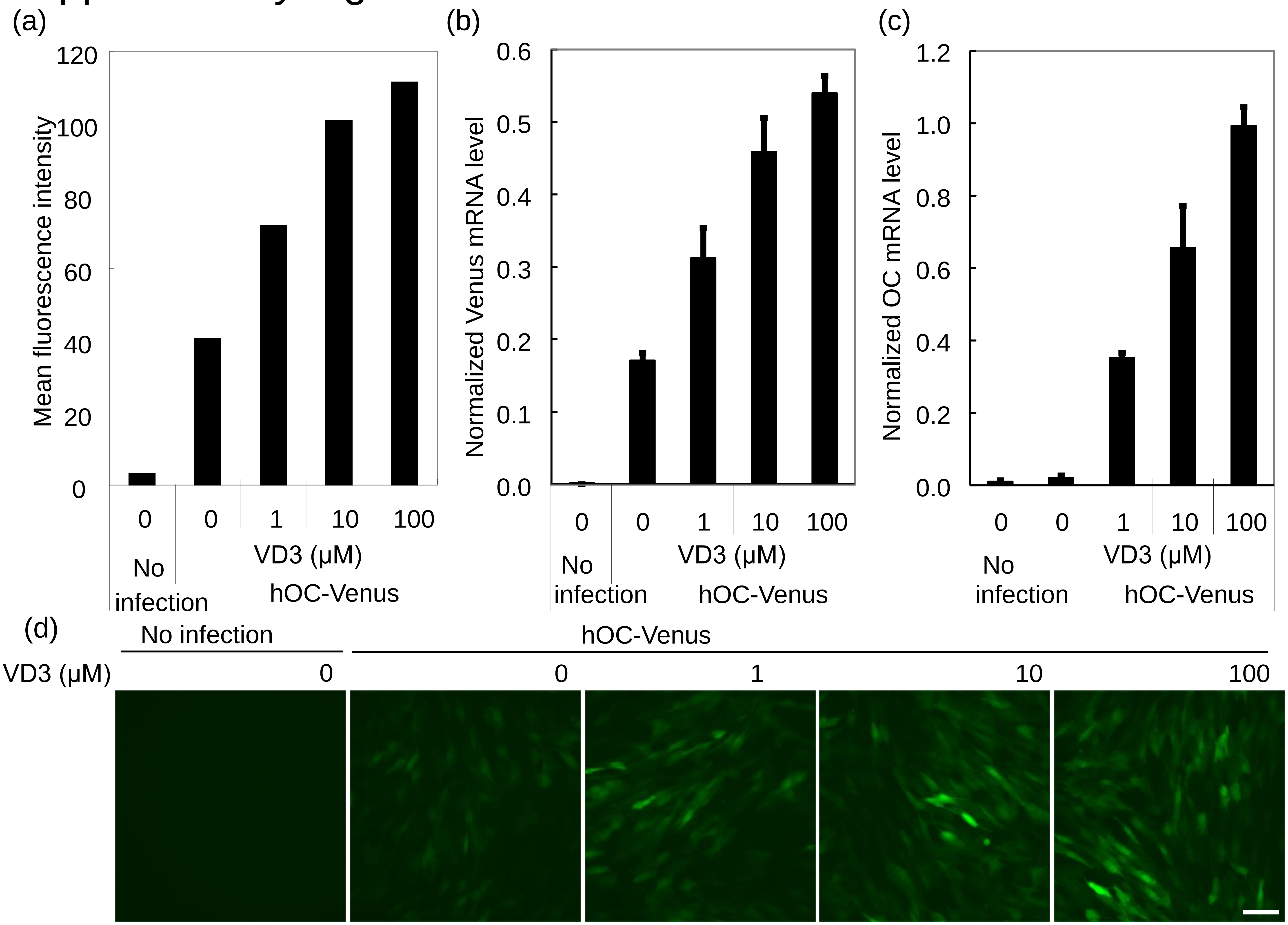

Supplementary Fig. S5

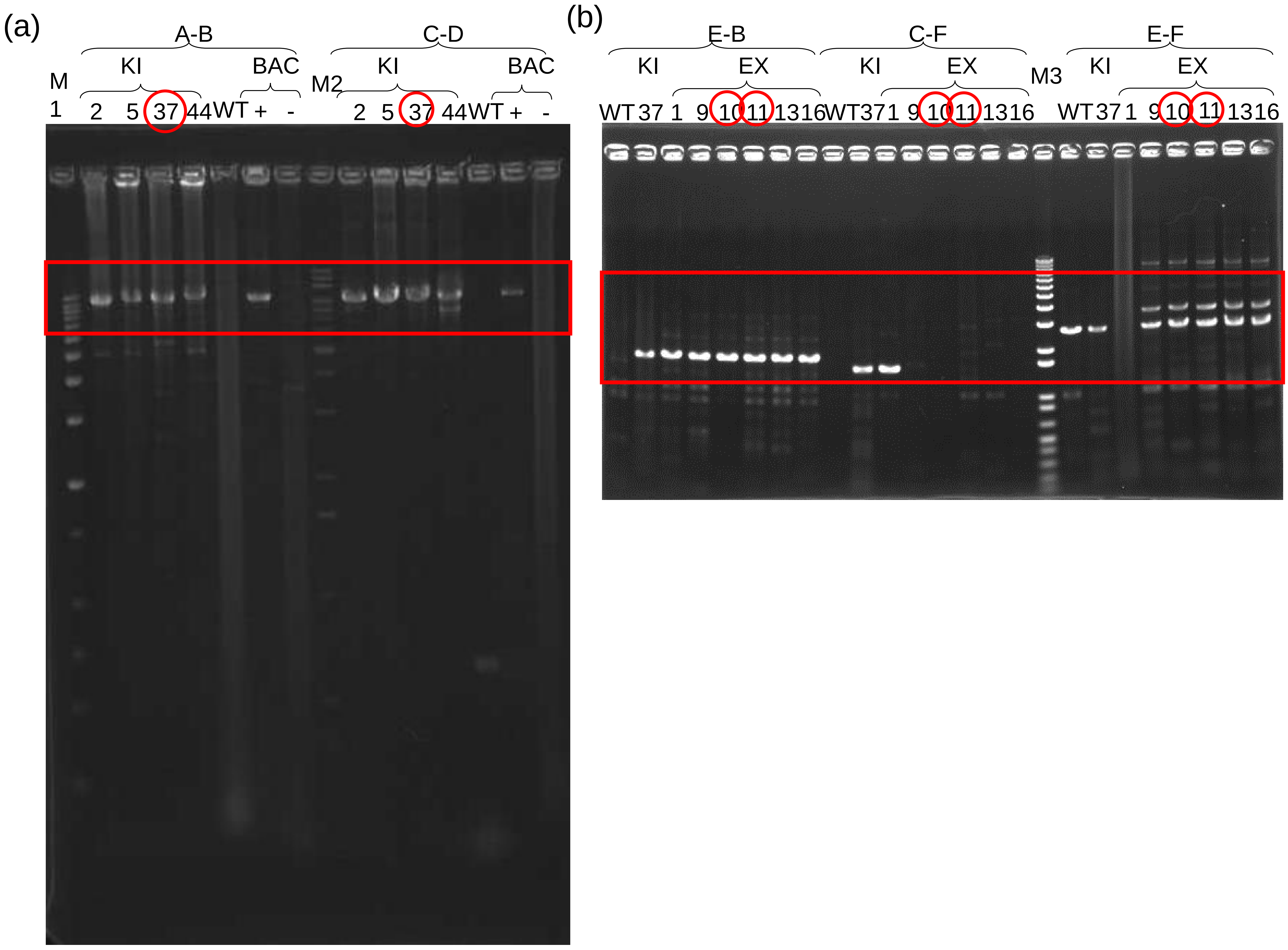

Supplementary Fig. S6

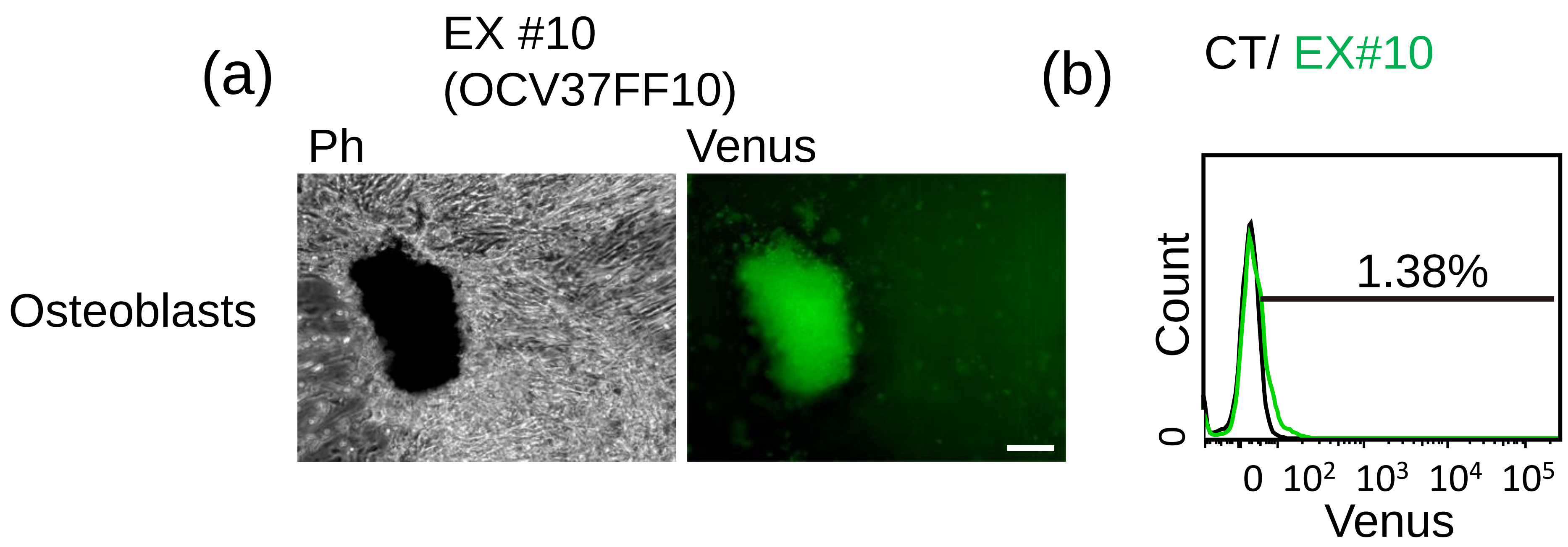

Supplementary Fig. S7

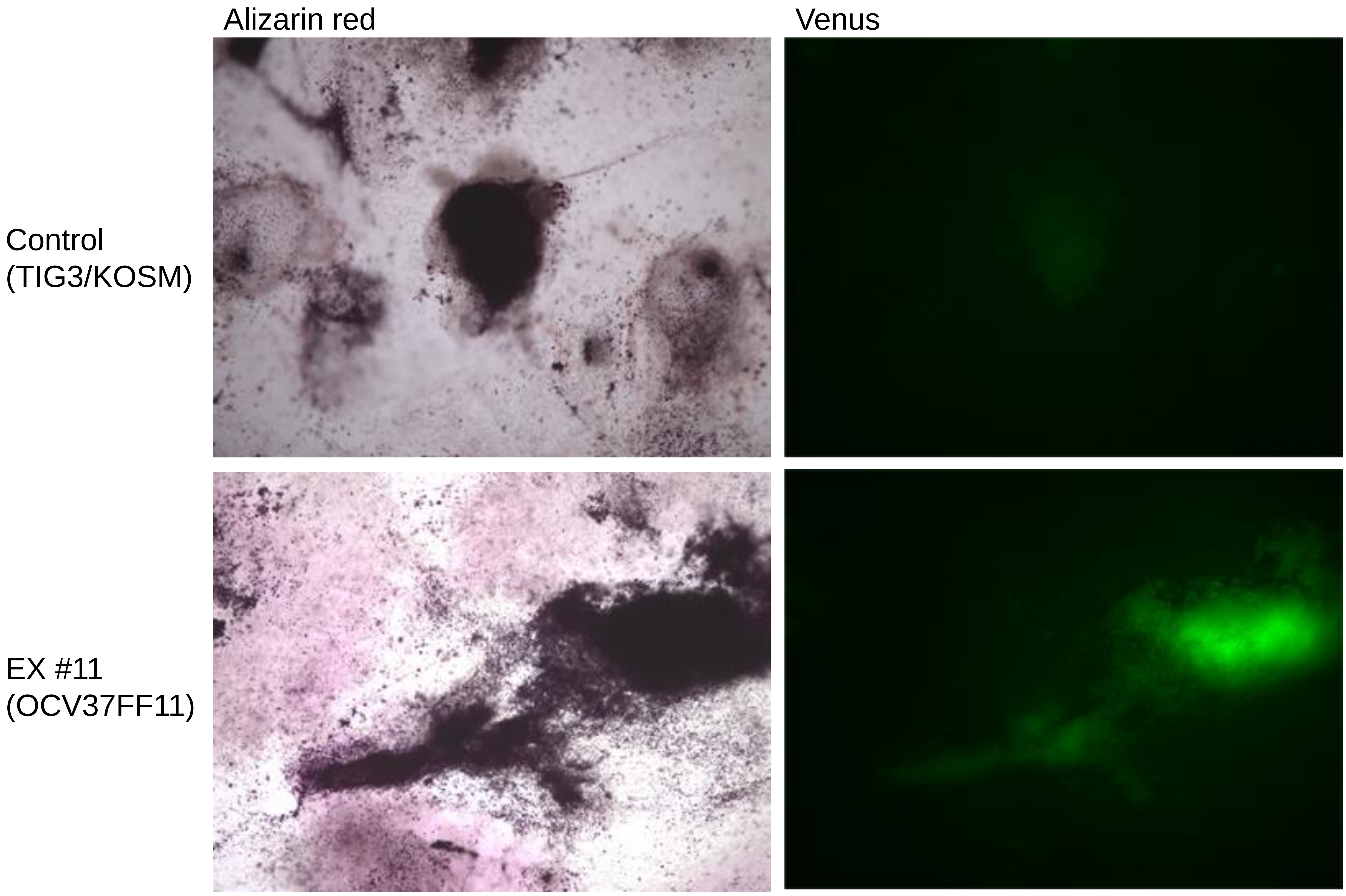

Supplementary Fig. S8

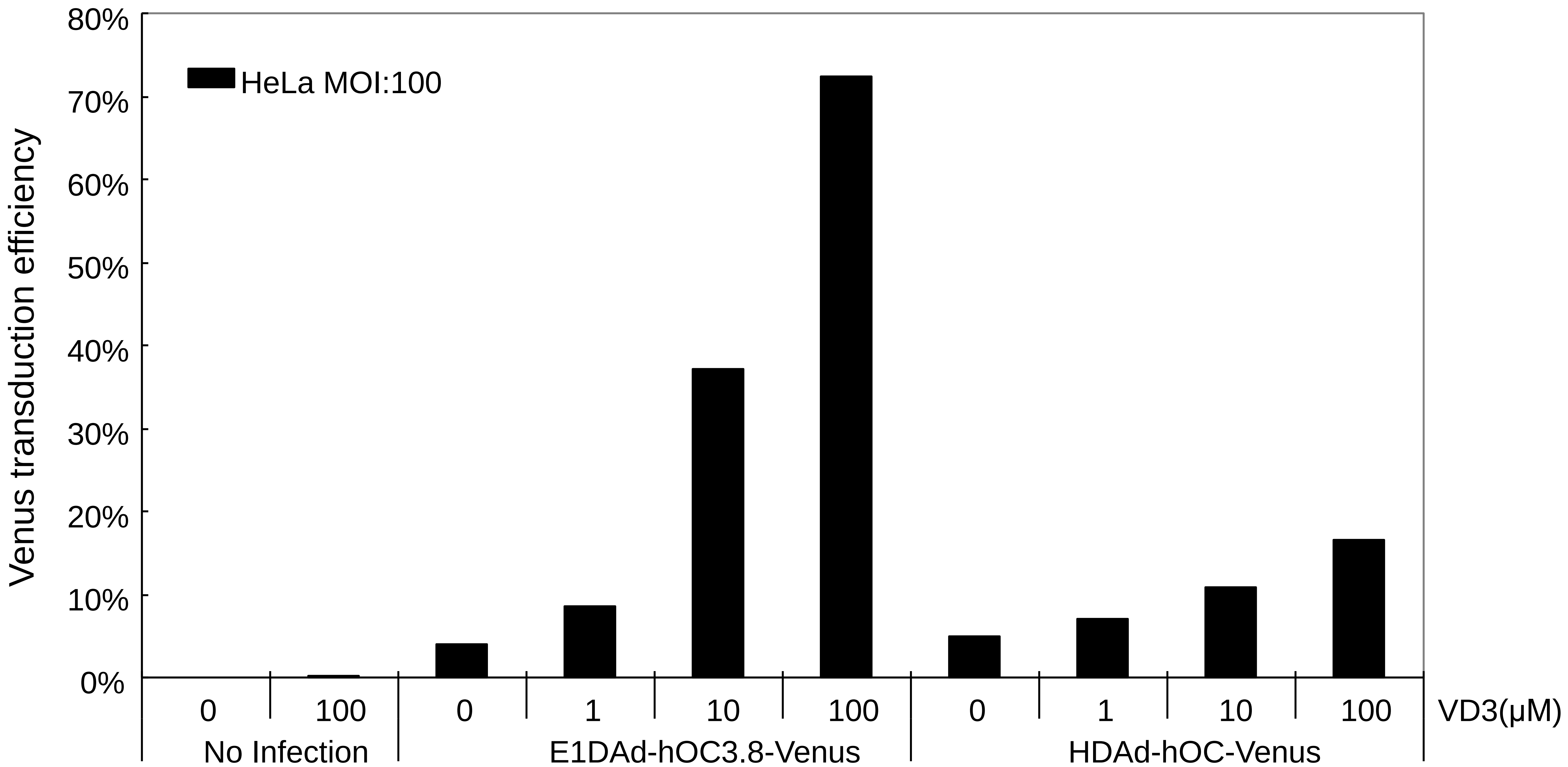

Supplementary Fig. S9

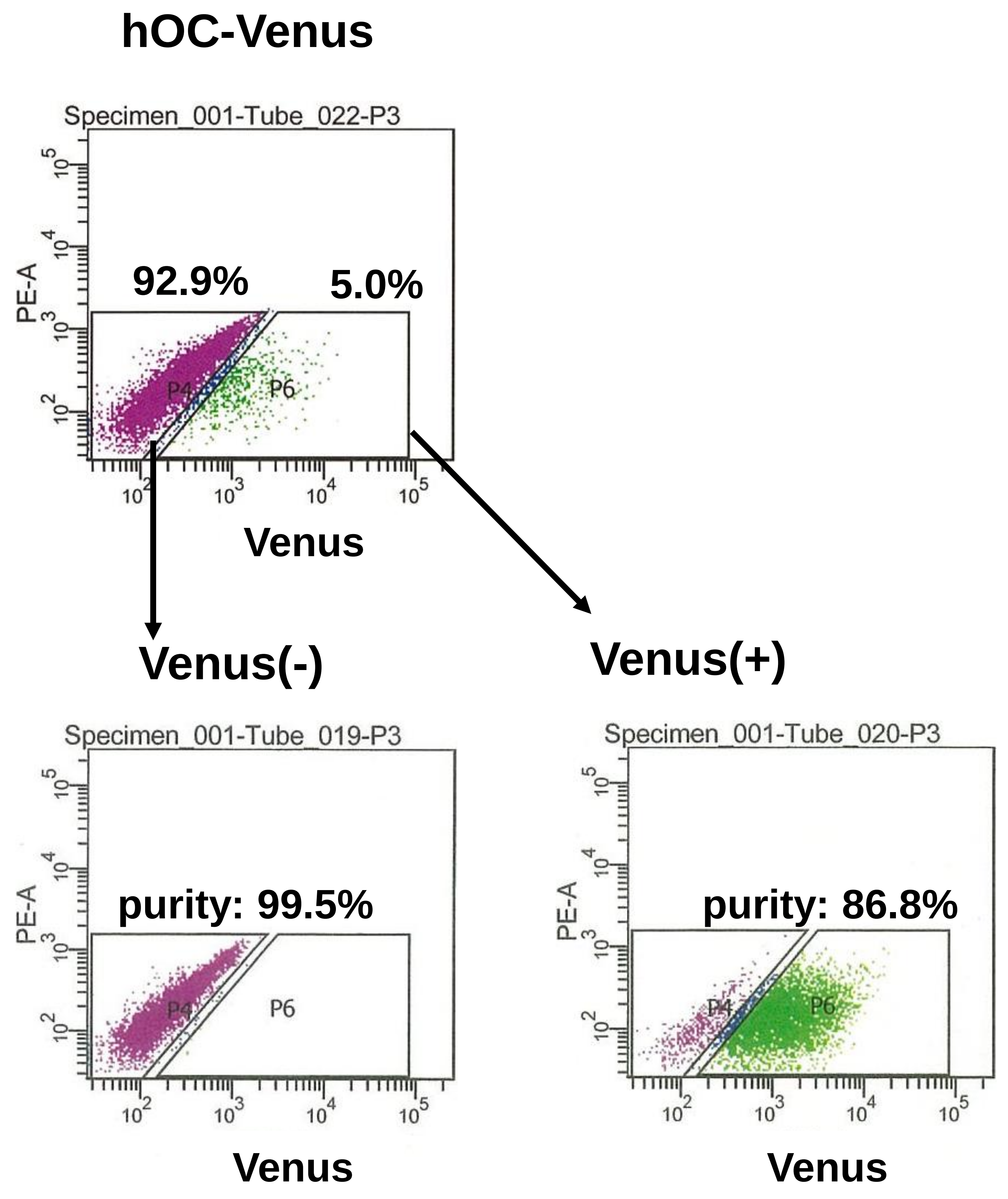

Supplement: Supplementary file 1 — Supplementary Methods, Supplementary Figure Legends 1-9, Supplementary Table 1 [file 41598_2019_46105_MOESM1_ESM.pdf]
